# An Analysis of Waves Underlying Grid Cell Firing in the Medial Enthorinal Cortex

**DOI:** 10.1186/s13408-017-0051-7

**Published:** 2017-08-25

**Authors:** Mayte Bonilla-Quintana, Kyle C. A. Wedgwood, Reuben D. O’Dea, Stephen Coombes

**Affiliations:** 1 Centre for Mathematical Medicine and Biology, School of Mathematical Sciences, University of Nottingham, University Park, NG7 2RD Nottingham, UK; 2 Centre for Biomedical Modelling and Analysis, University of Exeter, Living Systems Institute, Stocker Road, EX4 4QD Exeter, UK

**Keywords:** Grid cell, Medial enthorinal cortex, h-current, Rebound spiking, Integrate-and-fire neural field model, Nonsmooth dynamics, Travelling wave, Evans function

## Abstract

Layer II stellate cells in the medial enthorinal cortex (MEC) express hyperpolarisation-activated cyclic-nucleotide-gated (HCN) channels that allow for rebound spiking via an $I_{\text{h}}$ current in response to hyperpolarising synaptic input. A computational modelling study by Hasselmo (Philos. Trans. R. Soc. Lond. B, Biol. Sci. 369:20120523, 2013) showed that an inhibitory network of such cells can support periodic travelling waves with a period that is controlled by the dynamics of the $I_{\text{h}}$ current. Hasselmo has suggested that these waves can underlie the generation of grid cells, and that the known difference in $I_{\text{h}}$ resonance frequency along the dorsal to ventral axis can explain the observed size and spacing between grid cell firing fields. Here we develop a biophysical spiking model within a framework that allows for analytical tractability. We combine the simplicity of integrate-and-fire neurons with a piecewise linear caricature of the gating dynamics for HCN channels to develop a spiking neural field model of MEC. Using techniques primarily drawn from the field of nonsmooth dynamical systems we show how to construct periodic travelling waves, and in particular the dispersion curve that determines how wave speed varies as a function of period. This exhibits a wide range of long wavelength solutions, reinforcing the idea that rebound spiking is a candidate mechanism for generating grid cell firing patterns. Importantly we develop a wave stability analysis to show how the maximum allowed period is controlled by the dynamical properties of the $I_{\text{h}}$ current. Our theoretical work is validated by numerical simulations of the spiking model in both one and two dimensions.

**Electronic Supplementary Material:**

The online version of this article (doi:10.1186/s13408-017-0051-7) contains supplementary material.

## Introduction

The ability to remember specific events occurring at specific places and times plays a major role in our everyday life. The question of how such memories are established remains an active area of research, but several key facts are now known. In particular, the 2004 discovery of grid cells in the medial enthorinal cortex (MEC) by Fyhn et al. [2], supports the notion of a cognitive map for navigation. This is a mental representation whereby individuals can acquire, code, store, recall, and decode information about relative spatial locations in their environment. The concept was introduced by Tolman in 1948 [3], with the first neural correlate being identified as the place cell system in the hippocampus [4]. *Place cells* are found in the hippocampus and fire selectively to spatial locations, thereby forming a place field whose properties change from one environment to another. More recently, a second class of cells was identified that fire at the nodes of a hexagonal lattice tiling the surface of the environment covered by the animal—these are termed *grid cells* [5]. As an animal approaches the centre of a grid cell firing field, the spiking output of grid cell will increase in frequency. The grid field size and spacing increases from dorsal to ventral positions along the MEC and is independent of the animal’s speed and direction (even in the absence of visual input) and independent of the arena size. In rats, the grid field spacing can range from roughly 30 cm up to several meters [6]. Other grid cell properties include firing field patterns that manifest instantly in novel environments and maintain alignment with visual landmarks. Furthermore, neighbouring grid cells have firing fields with different spatial phases, whilst grid cells with a common spacing also have a common orientation (overturning an original suggestion that they have different orientations) [7].

May-Britt Moser and Edvard Moser shared the 2014 Nobel Prize in Physiology or Medicine with John O’Keefe for their discoveries of cells (grid and place cells, respectively) that subserve the brain’s internal positioning system. From a modelling perspective grid cells have attracted a lot of attention, due in part to their relatively recent and unexpected discovery, but also due to the very geometric firing patterns that they generate. There are now three main competing mathematical models for the generation of grid-like firing patterns: oscillatory interference models, continuous attractor network models, and “self-organised” models—see Giocomo et al. [8] and Schmidt-Hieber and Häusser [9] for excellent reviews. The first class of model uses interference patterns generated by multiple oscillations to explain grid formation [10]. They have been especially fruitful in addressing the theta rhythmic firing of grid cells (5–12 Hz) and their *phase precession*. Here spikes occur at successively earlier phases of the theta rhythm during a grid field traversal, suggestive of a spike-timing code [11]. The second class uses activity in local networks with specific connectivity to generate the grid pattern and its spacing. In this category, the models can be further sub-divided into those that utilise spatial pattern formation across the whole tissue (possibly arising via a Turing instability), such as in the work of Fuhs and Touretzky [12] and Burak and Fiete [13], and those that rely only upon spatially localised pattern states (or bumps) in models with (twisted) toroidal connectivity as described by McNaughton et al. [14] and Guanella et al. [15]. The third class proposes that grid cells are formed by a self-organised learning process that borrows elements from both former classes [16–18]. Recent experiments revealing the in vivo intracellular signatures of grid cells, the primarily inhibitory recurrent connectivity of grid cells, and the topographic organisation of grid cells within anatomically overlapping modules of multiple spatial scales along the dorsoventral axis of MEC provide strong constraints and challenges to all three classes of models [18–20]. This has led to a variety of new models, each with a focus on one or more aspects of biophysical reality that might underlie the functionality of grid cell response. For example Couey et al. [21] have shown that a continuous attractor network with pure inhibition can support grid cell firing, with the caveat that there is sufficient excitatory input to the MEC, supposedly from hippocampus, to cause principal cells to fire. However, recent optogenetic and electrophysiological experiments have challenged this simple description [22], highlighting the importance of intrinsic nonlinear ionic currents and their distribution amongst the main cell types in MEC.

Stellate and pyramidal cells constitute the principal neurons in layer II of medial enthorinal cortex (MEC II) that exhibit grid cell firing. The former comprise approximately 70% of the total MEC II neural population and are believed to represent the majority of the grid cell population. Even before the discovery of grid cells stellate cells were thoroughly studied because of their rapid membrane time constants and resonant behaviour. Indeed, they are well known to support oscillations in the theta frequency range [23, 24]. Interestingly the frequency of these intrinsic oscillations decreases along the dorsal-ventral axis of MEC II [25], suggestive of a role in grid field spacing. The resonant properties of stellate cells have been directly linked to a high density of hyperpolarisation-activated cyclic-nucleotide-gated (HCN) channels [26], underlying the so-called $I_{\text{h}}$ current. The time constant of both the fast and slow component of $I_{\text{h}}$ is significantly faster for dorsal versus ventral stellate cells, providing a potential mechanism for the observed difference in the resonant frequency along the dorsal-ventral axis. However, perhaps of more importance is the fact the $I_{\text{h}}$ current can cause a depolarising rebound spike following a hyperpolarising current injection. Given that stellate cells are mainly interconnected by inhibitory interneurons [21], this means that rebound can play an important role in shaping spatio-temporal network rhythms. The inclusion of important intrinsic biophysical properties into a network has been emphasised by several authors, such as Navratilova et al. [27] regarding the contribution of after-spike potentials of stellate cells to theta phase precession, and perhaps most notably by Hasselmo and colleagues for the inclusion of HCN channels [28–31]. This has culminated in a spiking network model of MEC that supports patterns whose periodicity is controlled by a neuronal resonance frequency arising from an $I_{\text{h}}$ current [1]. The model includes many of the features present in the three classes described above, and is able to replicate behaviour from several experiments, including phase precession in response to a phasic medial septum input, theta cycle skipping, and the loss of the spatial periodicity of grid cell firing fields upon a reduction of input from the medial septum. Simulations of the model in one spatial dimension show that the spacing of grid firing fields can be controlled by manipulating the speed of the rebound response. We note that a change that affects the rebound response would also affect the resonant properties of the cell. In contrast to continuous attractor models that rely on the spatial scale of connectivity to control grid spacing, a change in rebound response provides a mechanism of *local* control via changes in the expression levels of HCN channels. This fascinating observation warrants a deeper mathematical analysis. In this paper we introduce a spiking network model that shares many of the features of the Hasselmo model [1], focussing on the formation of travelling waves that can arise in the absence of (medial septum) input. Importantly our bespoke model is built from piecewise linear and discontinuous elements that allows for an explicit analysis of the periodic waves that arise in an inhibitory network through rebound spiking. In particular our wave stability analysis demonstrates clearly that the maximum allowed period is strongly controlled by the properties of our model $I_{\text{h}}$ current. This gives further credence to the hypothesis that HCN channels can control the properties of tissue level periodic waves that may underpin the spacing of grid cell firing in MEC.

In Sect. 2 we introduce our model of a network of stellate cells in MEC II. The single neuron model is a simple leaky integrate-and-fire (LIF) model with the inclusion of a synaptic current mediated by network firing events, and a model of $I_{\text{h}}$ based on a single gating variable. For ease of mathematical analysis we focus on a continuum description, so that the model may be regarded as a *spiking neural field*. Simulations of the model in two spatial dimensions are used to highlight the genericity of spike rebound mediated spatio-temporal patterns. To uncover the systematic way in which a cellular $I_{\text{h}}$ current can control the properties of patterns at the tissue level, we focus next on a one-dimensional version of the model without external input. By further developing a piecewise linear (pwl) caricature of the activation dynamics of a HCN channel we show in Sect. 3 how explicitly to construct the dispersion curve for periodic travelling waves. This gives the speed of a wave as a function of its period, and shows the possibility of a wide range of wave periods that could be selected. Next in Sect. 4 we exploit techniques from the analysis of nonsmooth systems to determine the Evans function for wave stability. Importantly an investigation of this function shows that the maximum allowed period can be controlled both by the overall conductance strength of the $I_{\text{h}}$ current as well as the time-scale for activation of HCN channels. Finally in Sect. 5 we discuss natural extensions of our work, and its relevance to further models of grid cell firing.

## The Model

The original work of Hasselmo [1] considered both simple resonant models as well as spiking nonlinear integrate-and-fire Izhikevich units to describe MEC stellate cells. The former incorporated a model for $I_{\text{h}}$ using a single gating variable with a linear dynamics whilst the latter were tuned to capture the resonant and rebound spiking properties from experimental data. Synaptic interactions between cells in a discrete one-dimensional network were modelled with a simple voltage threshold process. These models were subsequently studied in more detail in [28], with a further focus on two-dimensional models and travelling waves. Here, we consider a biophysically realistic spiking model in a similar spirit to that of Hasselmo, but within a framework that will allow for a subsequent mathematical analysis. In particular, we consider a spiking model of stellate cells using a generalised LIF model that includes a nonlinear $I_{\text{h}}$ current. Moreover, we opt for a description of synaptic interactions using an event-based scheme for modelling post-synaptic conductances.

We first consider a continuum description defined on the plane and introduce a voltage variable $V=V(\boldsymbol {r},t)$, where $\boldsymbol {r} \in \mathbb{R}^{2}$ and $t \geq0$. The subthreshold LIF dynamics is given by

1 $$ C \frac{\partial}{\partial t} V (\boldsymbol {r},t) = - g_{\text{l}} V (\boldsymbol {r},t) + I_{\text{h}} (\boldsymbol {r},t) + I_{\text{syn}} (\boldsymbol {r},t) + I_{\text{hd}} (\boldsymbol {r},t), $$

with a set of spike times at position ***r*** generated according to

2 $$ T^{m}(\boldsymbol {r}) = \inf\bigl\{ t \mid V(\boldsymbol {r},t) \geq V_{\text{th}} ; t \geq T^{m-1}(\boldsymbol {r}) + \tau_{\text{R}} \bigr\} , \quad m \in \mathbb{Z}. $$

Here $\tau_{\text{R}}$ is a refractory time-scale. Upon reaching the threshold $V_{\text{th}}$ the membrane potential is reset to the value $V_{\text{r}} < V_{\text{th}}$. The infimum operation ensures that a firing time is determined by the first time that the voltage variable (at a fixed position) crosses threshold (from below, remembering the IF reset process) subject to refractoriness. To model the refractory process we hold the voltage variable at the reset value $V_{\text{r}}$ for a duration $\tau_{\text{R}}$ after a firing event. The left-hand side of (1) describes a membrane current with capacitance *C*. The first term on the right-hand side of (1) represents a leak with a constant conductance $g_{l}$ (and we have set the leakage reversal potential to zero without loss of generality). The terms $I_{\text{h}}$, $I_{\text{syn}}$, and $I_{\text{hd}}$ represent currents arising from HCN channels, synaptic input, and head-direction input, respectively. $I_{\text{h}}$ is a slow inward current with a reversal potential $V_{\text{h}}$ that is substantially above resting levels, but which requires hyperpolarisation to become active; that is, the activation curve is monotone decreasing in *V*. Furthermore, the $I_{\text{h}}$ current does not inactivate, even with prolonged (minutes) hyperpolarisation. Thus it is often modelled with a single gating variable $n_{\text{h}}$ such that $I_{\text{h}} (\boldsymbol {r},t)= g_{\text{h}} n _{\text{h}} (\boldsymbol {r},t) (V_{\text{h}}-V (\boldsymbol {r},t))$, where

3 $$ \tau_{\text{h}}(V) \frac{\partial}{\partial t} n_{\text{h}} = n_{\text{h},\infty}(V) - n_{\text{h}} . $$

Here the shape for the activation function $n_{\text{h},\infty}$ is the sigmoid:

4 $$ n_{\text{h},\infty}(V)= \frac{1}{1+\exp((V-V_{1/2})/k)}, $$

and fits to experimental data give $V_{1/2}\approx-10\mbox{ mV}$ (with respect to rest) and $k\approx10$ [32, 33]. The time constant for activation and deactivation can vary from tens to hundreds of milliseconds [34]; however, here we fix $\tau_{\text{h}}=\text{constant}$ for simplicity and ignore any detailed dependence on voltage. To model synaptic interactions we consider a simple effective anatomical model whereby stellate cells interact directly through inhibition. In reality inhibitory interactions are actually mediated by interneurons and there is no direct synaptic coupling between stellate cells. Introducing an overall strength of synaptic conductance $g_{\text{syn}}$ we then write $I_{\text{syn}}(\boldsymbol {r},t)=g_{\text{syn}} \psi(\boldsymbol {r},t)$, where

5 $$ \psi(\boldsymbol {r},t) = \int_{\Omega} \mathrm {d}\boldsymbol {r}' W\bigl(\boldsymbol {r},\boldsymbol {r}'\bigr) E\bigl(\boldsymbol {r}', t\bigr), $$

where the function *W* represents anatomical connectivity, $\Omega \subseteq \mathbb{R}^{2}$ is the spatial domain, and the function *E* represents the shape of a post-synaptic response to a train of incoming spikes. We write this in the form

6 $$ E(\boldsymbol {r},t) = \sum_{m \in \mathbb{Z}} \eta\bigl(t-T^{m}( \boldsymbol {r})\bigr) , $$

for a given temporal filter shape *η* with the property that $\eta (t)=0$ for $t<0$ (so that interactions are causal). For convenience we will work with normalised responses such that $\int_{0}^{\infty} \mathrm {d}t \eta(t) = 1$. As a concrete choice for the function *W* we shall take a smoothed bump shape $W(\boldsymbol {r},\boldsymbol {r}')=w(|\boldsymbol {r}-\boldsymbol {r}'|)$, with

7 $$ w(x) = \frac{w_{0}}{2} \bigl[ \tanh\bigl(\beta(\sigma-x)\bigr) + \tanh\bigl( \beta (\sigma+x)\bigr) \bigr] , \quad\beta,\sigma>0 . $$

**Fig. 1:**
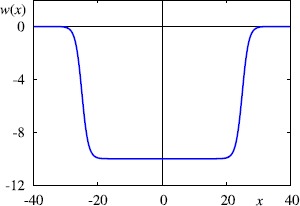
Connectivity function *w* in (7), with $\sigma= 25$, $\beta= 0.5$ and $w_{0} = -10$

Here $w_{0}<0$ in accordance with the predominantly inhibitory interactions of MEC, *σ* controls the spatial scale of interaction, and *β* the steepness of the surround inhibition function as shown in Fig. 1. The model is completed with the choice of the synaptic filter *η*, which we shall take to be an *α*-function of the form

8 $$ \eta(t) =\alpha^{2} t \mathrm {e}^{-\alpha t} H(t), $$

where $\alpha^{-1}$ is the time-to-peak of the synapse, and *H* is the Heaviside step function. Note that we work in a regime where the model is *excitable*, as we do not include any background drive that would be able to make the single neuron model fire in the absence of synaptic coupling or head-direction input.

An animal’s speed $v=v(t)$ and direction of motion $\varPhi=\varPhi(t)$ generates input to the MEC that is modelled by the head-direction current $I_{\text{hd}}$. For example, this could be of the form $I_{\text{hd}}(\boldsymbol {r},t)= \boldsymbol {v} \cdot \boldsymbol {r}_{\phi}$ where $\boldsymbol {v} = v(\cos{\varPhi}, \sin{\varPhi})$ and $\boldsymbol {r}_{\phi}= l(\cos\phi(\boldsymbol {r}), \sin\phi(\boldsymbol {r}))$ describes a head-direction preference for the orientation $\phi(\boldsymbol {r})$ at position ***r*** [13]. Here *l* is a constant that sets the magnitude of the head-direction current. The hidden assumption here is that head direction matches the direction of motion. However, this is not necessarily true behaviourally, and head direction cells may not code for motion direction [35]. Nonetheless it is a common assumption in most grid cell models, and so we adopt it here too. Fuhs and Touretzky [12] have shown that choosing an anisotropic anatomical connectivity of the particular form $W(\boldsymbol {r},\boldsymbol {r}') = w(|\boldsymbol {r}-\boldsymbol {r}_{\phi}-\boldsymbol {r}'|)$ can then induce a spatio-temporal network pattern to flow in accordance with the pattern of head-direction information generated when traversing an environment. For continuous attractor network models that can generate, via a Turing instability, static hexagonal patterns with regions of high activity at the nodes of a triangular lattice, the induced movement of these *hot-spots* over a given point in the tissue gives rise to grid-like firing patterns. In this case the spacing of the firing field is hard to change, as it is mainly fixed by the spatial scale of the chosen connectivity; however, the mechanism of inducing pattern flow is robust to how the tissue pattern is generated. Thus, given the established success of the Fuhs and Touretzky mechanism we will not focus on this here, and instead turn our attention to the formation of relevant tissue patterns and, in particular, how local control of firing field spacing may be effected.

**Fig. 2:**
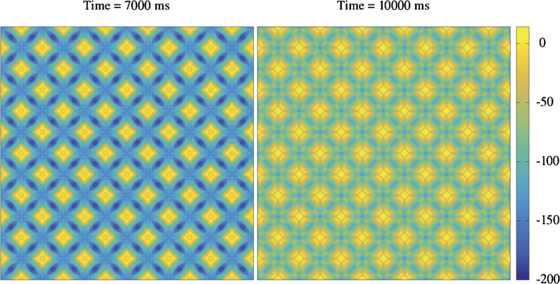
A simulation of spatially periodic non-travelling patterns in a two-dimensional spiking neural field model with an $I_{\mathrm{h}}$ current, solved on a spatial grid of 1000 × 1000 points. Displayed is the voltage component across the entire network at $t=7000\mbox{ ms}$ (*left*) and $t=10\mbox{,}000\mbox{ ms}$ (*right*). The model supports periodic patterns of localised activity. Note that these patterns are not static, but oscillate in time. Parameters: $C = 1\mbox{ }\upmu \mbox{Fcm}^{-2}$, $\tau_{\text{h}}=400\mbox{ ms}$, $V_{\text{h}}=40 \mbox{ mV}$, $g_{l} = 0.25\mbox{ mS/cm}^{-2}$, $g_{h} = 1\mbox{ mS/cm}^{-2}$, $\tau_{\mathrm{R}} = 200\mbox{ ms}$, $V_{\text{th}} = 14.5\mbox{ mV}$, $V_{\text{r}} = 0\mbox{ mV}$, $V_{1/2} = -10\mbox{ mV}$, $k=10$, $g_{\text{syn}} = 15\mbox{ mS/cm}^{-2}$, $w_{0} = -10$, $\sigma= 25$, $\beta ^{-1}=0$, and $\alpha^{-1} = 20\mbox{ ms}$. The choice of a long refractory time-scale in the model is useful for eliciting a single (rather than multiple) spike rebound response. Spatial domain $\Omega= [-L,L] \times[-L,L]$ where $L=10 \sigma$. See also the video in Additional file 2, showing the emergence of more exotic spatio-temporal structures, including hexagons

**Fig. 3:**
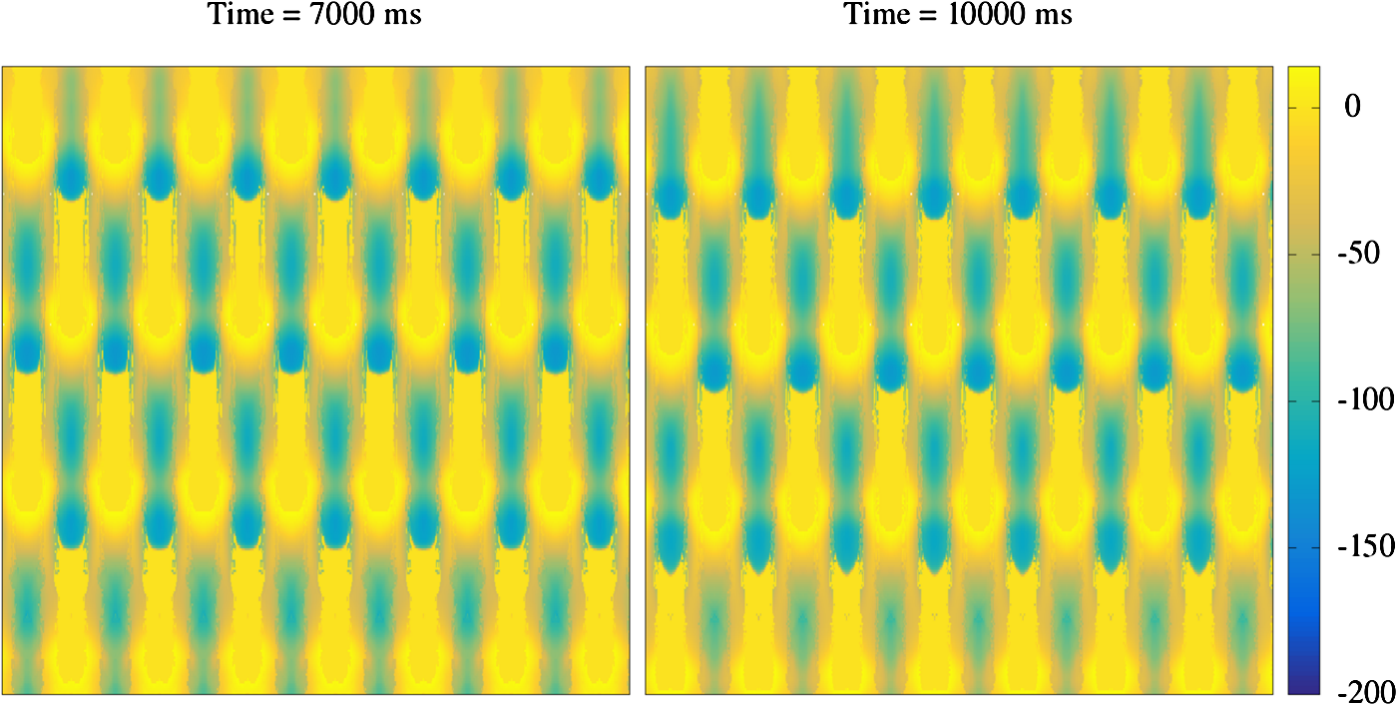
Periodic travelling wave solutions in the spiking neural field model, solved on a spatial grid of 1000 × 1000 points. Displayed is the voltage component across the entire network at $t=7000\mbox{ ms}$ (*left*) and $t=10\mbox{,}000\mbox{ ms}$ (*right*). Using different initial data, the spatially periodic pattern observed in Fig. 2 is replaced by a periodic travelling wave (moving downward in the figure). Parameters as in Fig. 2, with $w_{0}=-0.1$

To investigate the types of solution the spiking neural field model supports, we perform simulations over a two-dimensional square domain. Since the action of the head-direction current is merely to induce a flow of emergent patterns we restrict our attention to the case without such input, i.e. $I_{\text{hd}}=0$. See Appendix A for details of our bespoke numerical scheme implemented on a GPU architecture, and Additional file 1 for C^++^/CUDA code. We observe three general classes of coherent behaviour that take the form of spatially periodic non-travelling structures (though which oscillate in time), travelling periodic waves, and *lurching* waves. The latter are also generically found in neural systems with rebound currents, such as in models of thalamic slices [36–38]. Unlike traditional smoothly propagating waves, which exhibit a stationary profile in a co-moving frame, lurching waves consist of patterns of activity in a localised region of the domain, which after some period of time, decay and an adjacent region of the domain becomes active. These waves appear to ‘lurch’ across the domain. Whilst interesting in their own right, we focus in this article on the analysis of smoothly propagating periodic waves, since these have been suggested by Hasselmo [1] to play a dominant role in the formation of grid-like firing patterns in MEC. We show an example of a non-travelling periodic structure in Fig. 2, at two different time points to illustrate that the pattern is not static, but oscillates in time. An example of a smoothly propagating travelling wave is shown in Fig. 3. The model also supports more exotic spatio-temporal structures, including hexagonal patterns, and for a movie showing a dynamic state with a hexagonal sub-structure see Additional file 2.

## Wave Construction

To understand more fully how $I_{\text{h}}$ controls the emergent scale of periodic waves seen in the simulations of Sect. 2 we now turn to a one-dimensional version of the model defined on the infinite domain. As in Sect. 2, we consider $I_{\text{hd}}=0$, in which case the model is isotropic and (5) reduces to

9 $$ \psi(x,t) = \sum_{m \in \mathbb{Z}} \int_{-\infty}^{\infty} \mathrm {d}y w\bigl(|x-y|\bigr) \eta\bigl(t-T^{m}(y) \bigr), \quad x \in \mathbb{R}, t>0 . $$

**Fig. 4:**
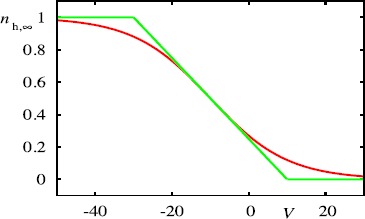
*Red line*: Nonlinear activation function for $n_{\text{h}}$ with $V_{1/2}=-10\mbox{ mV}$ and $k=10$. *Green line*: Piecewise linear fit of $n_{\text{h},\infty}$ given by Eq. (10)

To reduce the model to a more mathematically convenient form we make two observations about the $I_{\text{h}}$ current. The first is that $V_{\text{h}}$ is typically larger than *V*, which suggests the approximation $V_{\text{h}} - V \simeq V_{\text{h}}$. The second is that the nonlinear activation function $n_{\text{h},\infty}$ can be approximated by a pwl function, as illustrated in Fig. 4. Here we match the slope at $V=V_{1/2}$, and otherwise saturate the function to one or zero, so that

10 $$ n_{\text{h},\infty}(V) = \textstyle\begin{cases} 1 , & V \leq V_{-}\\ \frac{1}{2} - \frac{V-V_{1/2}}{4 k} , & V_{-} < V < V_{+}\\ 0 , & V \geq V_{+}, \end{cases}\displaystyle ,\quad V_{\pm}= V_{1/2} \pm2 k. $$

**Fig. 5:**
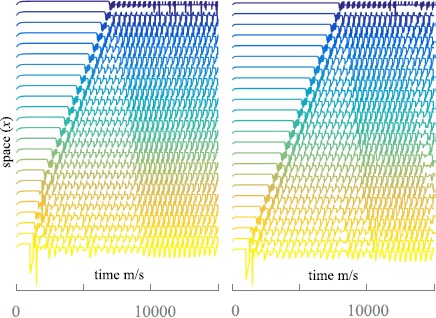
Simulations of a one-dimensional spiking neural field model with an $I_{\text{h}}$ current solved on a spatial grid of 5000 points. *Left*: simulations of the model with the full nonlinear dynamics for $I_{\text{h}}$. *Right*: simulations of the model with the reduced pwl dynamics for $I_{\text{h}}$ given by (10). Here we show the voltage traces at an illustrative set of locations throughout the system as a function of time. Note that each single spike is mediated by rebound in response to inhibitory synaptic input. Parameters are as in Fig. 2 with $\beta=0.5$, $V_{\text{th}} = 14\mbox{ mV}$. The activity propagates from bottom to top after an initial hyperpolarisation current of $-30\mbox{ mV}$ is given to a set of neurons (in *yellow*) from $t=1000\mbox{ ms}$ to $t=1250\mbox{ ms}$

**Fig. 6:**
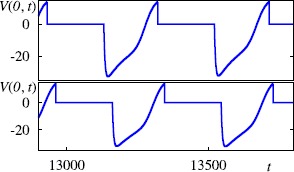
Voltage traces observed at $x=0$, when the periodic travelling wave is fully developed. *Top*: simulations of the model with the reduced pwl dynamics for $I_{\text{h}}$ given by (10). *Bottom*: simulations of the model with the full nonlinear dynamics for $I_{\text{h}}$. All parameters as in Fig. 5

Simulations of the model with the reduced form of the $I_{\text{h}}$ are in qualitative agreement with simulations of the full nonlinear model, and indeed wherever tested the same repertoire of behaviour is always found. In both instances, travelling wave behaviour with a well-defined speed and period is easily initiated; Figs. 5 and 6 compare simulation results arising in the fully nonlinear and reduced pwl model.

If we introduce the vector $X=(V,n_{\text{h}}) \in \mathbb{R}^{2}$ then we may write the reduced model in a more abstract setting, namely in terms of the pwl evolution equation that governs the system behaviour between one spiking event and the next:

11 $$ \frac{\partial}{\partial t} X(x,t) = A X(x,t) + \Psi(x,t) , \quad T^{m-1}(x) \leq t < T^{m}(x). $$

In (11), *A* is a $2 \times2$ matrix that is defined in a piecewise constant fashion, with dependence on the value of the voltage (in particular, which of the three domains detailed in equation (10) pertains), or whether the system is in the refractory state. In the latter case, *A* is defined according to

12 $$ A= A_{\text{R}} = \begin{bmatrix} 0 & 0\\ 0 & -1/\tau_{\text{h}} \end{bmatrix} , \quad T^{m-1}(x) \leq t < T^{m-1}(x)+\tau_{\text{R}}, $$

while for $T^{m-1}(x) + \tau_{\text{R}} \leq t < T^{m}(x)$

13 $$ A= \textstyle\begin{cases} A_{0} = \Bigl[{\scriptsize\begin{matrix}{} -1/\tau& g_{\text{h}} g_{\text{l}}^{-1}/\tau\cr -1/(4 k \tau_{\text{h}}) & -1/\tau_{\text{h}} \end{matrix}}\Bigr], & V_{-} < V < V_{+}, \\ A_{-} = \Bigl[{\scriptsize\begin{matrix}{} -1/\tau& g_{\text{h}} g_{\text{l}}^{-1}/\tau\cr 0 & -1/\tau_{\text{h}} \end{matrix}}\Bigr] , & V \leq V_{-}, \\ A_{+} = A_{-}, & V \geq V_{+}, \end{cases} $$

where we have assumed the ordering $V_{-} < V_{\text{r}} < V_{+} < V_{\text{th}}$, introduced $\tau=C/g_{\text{l}}$, and absorbed the factor $V_{\text{h}}$ within $g_{\text{h}}$. Similarly we define Ψ according to

14 $$ \Psi= \Psi_{\text{R}} = \begin{bmatrix} 0 \\ (1/2-(V_{\text{r}}-V_{1/2})/(4k))/\tau_{\text{h}} \end{bmatrix} , \quad T^{m-1}(x) \leq t < T^{m-1}(x)+ \tau_{\text{R}}, $$

and, for $T^{m-1}(x) + \tau_{\text{R}} \leq t < T^{m}(x)$,

15 $$ \Psi= \frac{g_{\text{syn}} g_{\text{l}}^{-1}}{\tau} \psi \begin{bmatrix} 1\\ 0 \end{bmatrix} + \textstyle\begin{cases} b_{0} , & V_{-} < V < V_{+}, \\ b_{-} , & V \leq V_{-}, \\ b_{+} , & V \geq V_{+}, \end{cases} $$

where

16 $$ b_{0} = \begin{bmatrix} 0\\ (1/2+V_{1/2}/(4k))/\tau_{\text{h}} \end{bmatrix} , \qquad b_{-}= \begin{bmatrix} 0\\ 1/\tau_{\text{h}} \end{bmatrix} , \qquad b_{+}= \begin{bmatrix} 0\\ 0 \end{bmatrix} . $$

We highlight that in (12)–(16) we have introduced the subscripts $\{R,0,-,+ \}$ to indicate the state of the system, namely whether it is refractory (labelled by *R*) or is not refractory and has a voltage in the range $(V_{-},V_{+})$ (labelled by 0), $(-\infty,V_{-}]$ (labelled by −), $[V_{+},\infty)$ (labelled by +).

### Travelling Wave Analysis

We now seek travelling wave solutions of (11) of the form $\widehat{X}(\xi,t)$, where $\xi=t-x/c$ and *c* is the (constant) wave speed. In this case (11) transforms to

17 $$ \biggl(\frac{\partial}{\partial t} + \frac{\partial}{\partial\xi} \biggr) \widehat{X}(\xi,t) = A \widehat{X}(\xi,t) + \widehat{\Psi }(\xi,t). $$

A stationary travelling wave $\widehat{X}(\xi,t) = Q(\xi) = (V(\xi ),n_{\text{h}}(\xi))$ satisfies the travelling wave equation

18 $$ \frac{\mathrm {d}Q}{\mathrm {d}\xi} = A Q(\xi) + \widehat{\Psi}(\xi). $$

In terms of firing events a periodic wave is described by $T^{m}(x) = x/c + m \varDelta$, where *Δ* is the period of the wave such that $Q(\xi+\varDelta) = Q(\xi)$. Substitution of this firing ansatz into (9) allows us to determine the function $\widehat{\psi}(\xi ) = \psi(x,t) \vert _{t=T^{m}(x)}$ where $\widehat{\Psi}(\xi )$ is obtained from (15) under the replacement of *ψ* by *ψ̂*. The function *ψ̂* is easily determined as

19 $$ \widehat{\psi}(\xi) = c \sum_{m \in \mathbb{Z}} \int_{0}^{\infty} \mathrm {d}s \eta(s) w\bigl(\big|c(s-\xi) + c m \varDelta\big|\bigr) , $$

and is *Δ*-periodic, and can therefore be expressed in terms of a Fourier series as

20 $$ \widehat{\psi}(\xi) = \sum_{p \in \mathbb{Z}} \psi_{p} \mathrm {e}^{-2 \pi i p \xi/\varDelta}, \quad\psi_{p} = \frac{1}{\varDelta} \widetilde{w} \biggl(\frac{2 \pi p}{c \varDelta} \biggr) \widetilde{\eta} \biggl(-\frac{2 \pi p}{\varDelta} \biggr) . $$

In (20) tildes denote the Fourier integral representation, such that for a given function $a(x)$

21 $$ a(x) = \frac{1}{2 \pi} \int_{-\infty}^{\infty} \mathrm {d}k ~ \widetilde {a}(k) \mathrm {e}^{ikx}, \quad \widetilde{a}(k) = \int_{-\infty}^{\infty} \mathrm {d}x ~ a(x) \mathrm {e}^{-ikx} , $$

and we have made use of the result that $c \varDelta\sum_{m} \mathrm {e}^{ikcm \varDelta} = 2 \pi\sum_{p} \delta(k-2 \pi p/(c \varDelta))$.

For the bump function (7) and the *α*-function (8) we have

22 $$ \widetilde{w}(k) = w_{0} \frac{\pi}{\beta} \frac{\sin k \sigma }{\sinh(\pi k /(2 \beta))} , \qquad \widetilde{\eta}(k) = \frac{\alpha^{2}}{(\alpha+ik)^{2}} . $$

Thus, given the decay properties of (22) as a function of *k*, the sum in (20) can be naturally truncated.

The formal solution to (18) can be constructed using variation of parameters as

23 $$ Q(\xi) = G(\xi,\xi_{0}) Q(\xi_{0}) + \int_{\xi_{0}}^{\xi}G\bigl(\xi,\xi'\bigr) \widehat{\Psi}\bigl(\xi'\bigr) \,\mathrm {d}\xi', $$

where *G* is a matrix exponential given by

24 $$ G\bigl(\xi,\xi'\bigr) = \mathcal{T} \biggl\{ \exp \biggl( \int_{\xi'}^{\xi} \mathrm {d}s A(s) \biggr) \biggr\} . $$

Here $\mathcal{T}$ is a *time-ordering operator* $\mathcal {T} A(t) A(s) = H(t -s)A(t) A(s) + H(s-t) A(s)A(t)$. In general the issue of time-ordering makes it very difficult to evaluate *G*. However, in our case *A* is piecewise constant and so we easily may break the solution up into parts distinguished by the label $\mu\in\{R, 0, -,+\}$. In each case trajectories are given explicitly by (23) with $G(\xi,\xi')=G(\xi-\xi')$ and $G=G_{\mu}$ where $G_{\mu}(\xi) = \exp(A_{\mu}\xi)$. A global trajectory may then be obtained by patching together solutions, denoted by $Q_{\mu}$, from each domain. It is in this fashion that we now construct the shape of a periodic travelling wave in a self-consistent manner. Of use will be matrix exponential decomposition $\mathrm {e}^{A t} = P \mathrm {e}^{\Lambda t} P^{-1}$, where $\Lambda= \operatorname{diag} (\lambda^{+},\lambda^{-})$ are the eigenvalues of *A* with associated eigenvectors $q^{\pm}= (1, (\lambda^{\pm}- A_{11})/A_{12}, )^{T}$. Here the eigenvalues of *A* are given explicitly by

25 $$ \lambda^{\pm}= \frac{1}{2} \bigl( \operatorname{Tr}A \pm\sqrt {( \operatorname{Tr}A)^{2} - 4 \det A} \bigr) . $$

Using (20) we may then write a domain specific trajectory for $\mu\in\{0,+,-\}$ in the form

26 $$\begin{aligned}[b] Q_{\mu}(\xi) ={}& G_{\mu}(\xi-\xi_{0}) Q_{\mu}(\xi_{0}) +A_{\mu}^{-1} \bigl[G_{\mu}(\xi-\xi_{0}) -I_{2} \bigr] b_{\mu}\\ & + \frac{g_{\text{syn}} g_{\text{l}}^{-1}}{\tau} \sum_{p \in \mathbb{Z}} \psi_{p} P_{\mu}\operatorname{diag} \bigl( Z_{\mu}^{+}(\xi,\xi_{0}), Z_{\mu}^{-}(\xi, \xi_{0})\bigr) P_{\mu}^{-1} \begin{bmatrix} 1\\ 0 \end{bmatrix} ,\end{aligned} $$

where

27 $$ Z_{\mu}^{\pm}(\xi,\xi_{0}) = \frac{\mathrm {e}^{\lambda_{\mu}^{\pm}(\xi-\xi_{0})} \mathrm {e}^{-2 \pi i p \xi_{0}/\varDelta} - \mathrm {e}^{-2 \pi i p \xi/\varDelta }}{\lambda_{\mu}^{\pm}+2 \pi i p/\varDelta} . $$

Here $\Lambda_{\mu}= \operatorname{diag} (\lambda^{+}_{\mu},\lambda ^{-}_{\mu})$ with $\lambda^{\pm}_{\mu}$ representing the eigenvalues of $A_{\mu}$ and

28 $$ P_{\mu}= \begin{bmatrix} 1 & 1 \\ (\lambda^{+}_{\mu}- [A_{\mu}]_{11})/[A_{\mu}]_{12} & (\lambda^{-}_{\mu}- [A_{\mu}]_{11})/[A_{\mu}]_{12} \end{bmatrix} . $$

When $\mu=R$ we adopt an alternative strategy (since $A_{R}$ is singular), remembering that when the system is refractory then $V(\xi )$ is *clamped* at the value $V=V_{\text{r}}$. In this case, we only have to consider the evolution of the gating variable $n_{\text{h}}$, which is obtained from (3) and (10) to give

29 $$ n_{\text{h}}(\xi) = n_{\text{h}}(\xi_{0}) \mathrm {e}^{-(\xi-\xi_{0})/\tau_{\text{h}}} + \bigl(1/2-(V_{\text{r}}-V_{1/2})/(4k)\bigr) \bigl[1-\mathrm {e}^{-(\xi-\xi_{0})/\tau _{\text{h}}}\bigr] . $$

**Fig. 7:**
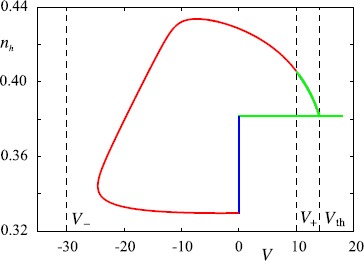
Orbit of a periodic travelling wave. Parameters as in Fig. 5, with $\varDelta= 450$, $c = 0.0669$, $n_{h}(0) = 0.3815$ and $\xi_{1} = 225.4223$. The travelling wave starts at $\xi=0$ where $V=V_{r}$ is clamped and $n_{h}$ evolves according to (29) until $\xi= \tau_{\mathrm{R}}$ and the system is released from the refractory period (*blue line*). Then it evolves clockwise according to (26) with $\mu= 0$ until $V(\tau_{\mathrm{R}} + \xi_{1})=V_{+}$ when it switches (*red line*) to $\mu=+$ (*green line*). The orbit ends when $V=V_{\text{th}}$ and *V* is reset. *The green horizontal line* for $V > V_{\text{th}}$ is not part of the solution. It is simply a marker for the spiking event (and the model does not generate an explicit shape for an action potential). *Black dotted lines* represent (from left to right) $V=V_{-}$, $V=V_{+}$ and $V=V_{\text{th}}$

**Fig. 8:**
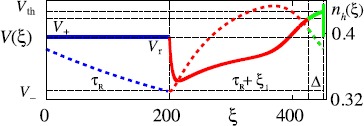
Profile of the two components of the periodic travelling wave $Q(\xi)$ defined by (26) and illustrated in Fig. 7. *Solid lines* correspond to $V(\xi)$ (*left-hand axis*) and *dotted* ones to $n_{h}(\xi)$ (*right-hand axis*). Colour-code and parameters as in Fig. 7. *Dotted black lines* indicate the values where the system changes dynamics

Now let us consider the form of a periodic wave which elicits a single spike for every period, much like the ones seen in Fig. 5. An example of such a travelling wave orbit in the $(V,n_{\text{h}})$ phase plane is shown in Fig. 7. The corresponding evolution of $V(\xi)$ and $n_{\text{h}}(\xi)$ is shown in Fig. 8.

**Fig. 9:**
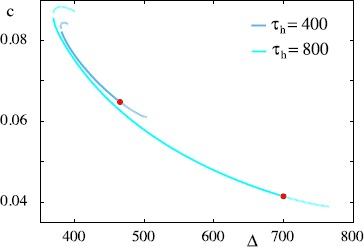
Dispersion curve $c=c(\varDelta)$ for a periodic travelling wave. Here *c* is the speed of a wave with period *Δ*. For small periods and on the upper branch waves are constructed that visit the domain where $V< V_{-}$. Parameters as in Fig. 5. *Solid lines* represent periods where the system is stable while *dashed lines* represent where it is unstable; *red dots* represent the maximum stable period of the orbit, highlighting its increase with $\tau_{h}$. Note that waves (on the lower branch of solutions) are stable for a large range of wave periods, and that the actual value of $(c,\varDelta)$ that would be observed in a simulation are dependent upon the choice of initial data

Given the translational invariance of the system we are free to choose a travelling wave origin such that $\xi=0$ corresponds to the system immediately after firing. For a duration $\tau_{\text{R}}$ it will then remain clamped at $V_{\text{r}}$ with $n_{\text{h}}$ evolving according to (29) with $\xi_{0}=0$ (as shown in blue in Fig. 7 and Fig. 8). From here it then evolves according to (26) with $\mu=0$, with initial data determined by $Q_{R}(\xi_{0})=(V_{\text{r}},n_{\text{h}}(\tau_{\text{R}}))$, until $V(\xi)$ reaches $V_{\pm}$, after which we set $\mu=-$ or $\mu =+$ in (26) and select appropriate initial data for (26), depending on the value of *V* achieved first. For simplicity, and since this is reliably observed in numerical simulations for a wide range of parameters (red line in Fig. 7 and Fig. 8, though the argument is easily generalised), we assume $V_{+}$ is the relevant choice. The final piece of the orbit is then obtained from (26) with $\mu=+$ and initial data determined by $Q_{+}(\xi_{0})=Q_{0}(\xi_{1}+\tau _{\text{R}})$ (green line in Fig. 7 and Fig. 8) and evolving the system until $V(\xi)=V_{\text{th}}$. Denoting the *time of flight* for the trajectory such that $V_{\text{r}} \leq V < V_{+}$ by $\xi_{1}$, and that, for $V_{+} \leq V < V_{\text{th}}$ by $\xi_{2}$, the period of the orbit is given by $\varDelta=\tau_{\text{R}} + \xi_{1} + \xi_{2}$. We note that the orbit is discontinuous because of the reset of the voltage variable after one period. Thus we have four unknowns $(n_{\text{h}}(0),c,\xi_{1},\varDelta)$ related by three nonlinear algebraic equations

30 $$ \textstyle\begin{cases} V(\varDelta)=V_{\text{th}} &\mbox{(firing condition),}\\ n_{\text{h}}(\varDelta)=n_{\text{h}}(0) &\mbox{(periodicity condition),}\\ V(\tau_{\text{R}}+\xi_{1})=V_{+} &\mbox{(switching condition),} \end{cases} $$

whose simultaneous solution determines the dispersion relationship for the wave speed as a function of the period $c=c(\varDelta)$. An example of a dispersion curve constructed in this way is shown in Fig. 9. Here we see that a wide range of allowed wavelengths can co-exist (with differing speeds). Note that in Fig. 9 we also include solutions that visit the region of phase-space where $V < V_{-}$, and these solutions typically only occur for small values of *Δ*. Our constructive theory does not provide a wave selection principle; however, by varying initial data in direct numerical simulations we were able to find solutions in excellent agreement with the theoretical predictions up to some maximal value of *Δ*. The determination of this value is the subject of the next section, where we show how to analyse wave stability.

## Wave Stability

To determine the stability of a periodic travelling wave we must not only treat perturbations of the state variables, but also the associated effects on the times of firing. Moreover, one must remember that because the model is nonsmooth (due to the switch at $V=V_{\pm}$) and discontinuous (because of reset whenever $V=V_{\text{th}}$) standard approaches for analysing smooth dynamical systems cannot be immediately applied. Nonetheless, as we show below, the wave stability can in fact be explicitly determined. We do this by constructing the so-called Evans function. This has a long history of use in the analysis of wave solutions to partial differential equations, dating back to the work of Evans on the stability of action potentials in the Hodgkin–Huxley model of a nerve fibre [39], has been extended to certain classes of firing rate neural field model [40], and is developed here for spiking neural fields.

We begin our analysis by exposing the spike-train that determines the synaptic drive in (9) by writing it in the equivalent form

31 $$ \psi(x,t) = \sum_{m \in \mathbb{Z}} \int_{-\infty}^{\infty} \mathrm {d}y w\bigl(|x-y|\bigr) \int_{-\infty}^{t} \mathrm {d}s \eta(t-s)\delta \bigl(s-T^{m}(y)\bigr), $$

where the firing times are defined according to the threshold condition $V(x,T^{m}(x)) = V_{\text{th}}$. We may relate spike times to voltage threshold conditions using the result that for fixed *x*

32 $$ \delta\bigl(t-T^{m}(x)\bigr) = \big|V_{t}\bigl(x,T^{m}(x) \bigr)\big| \delta\bigl(V(x,t)-V_{\text{th}}\bigr) , $$

and $V_{t}$ denotes partial differentiation of *V* with respect to *t*. Hence

33 $$\begin{aligned}[b] \psi(x,t) ={}& \sum_{m \in \mathbb{Z}} \int_{-\infty}^{\infty} \mathrm {d}y w\bigl(|x-y|\bigr)\\ &\times\int_{-\infty}^{t} \mathrm {d}s \eta(t-s) \big|V_{t} \bigl(y,T^{m}(y)\bigr)\big| \delta\bigl(V(y,s)-V_{\text{th}}\bigr) . \end{aligned} $$

Consider again travelling wave solutions of the form $V(x,t)=\widehat {V}(\xi,t)$, where $\xi=t-x/c$. In this co-moving frame we can define a set of firing event functions $\xi^{m}(t)$ according to the threshold condition $\widehat{V}(\xi^{m}(t),t)=V_{\text{th}}$. These event times can be related to the co-moving voltage threshold condition using the result that, for fixed *t*,

34 $$ \delta\bigl(\xi-\xi^{m}(t)\bigr) = \big|\widehat{V}_{\xi}\bigl( \xi^{m}(t),t\bigr)\big| \delta \bigl(\widehat{V}(\xi,t)-V_{\text{th}} \bigr) . $$

Substitution into (33) and using $\widehat{V}_{\xi}\simeq V_{t}$ close to a periodic orbit we find

35 $$\begin{aligned}[b] \psi(x,t) &= \sum_{m \in \mathbb{Z}} \int_{-\infty}^{\infty} \mathrm {d}y w\bigl(|x-y|\bigr) \int_{-\infty}^{t} \mathrm {d}s \eta(t-s) \delta\bigl(s-y/c- \xi^{m}(s)\bigr) \\ &= c \sum_{m \in \mathbb{Z}} \int_{0}^{\infty} \mathrm {d}s \eta(s) w\bigl(\big|c(s-\xi) + c \xi^{m} (t-s)\big|\bigr) \equiv\widehat{\psi}(\xi,t). \end{aligned} $$

Noting that, for a periodic wave $\xi^{m}(t) = m \varDelta$, *ψ̂* is independent of *t* and Eq. (35) recovers Eq. (19) as expected.

We now analyse the stability of such a periodic wave by perturbations such that $\xi^{m}(t) = m \varDelta+ \delta\xi^{m}(t)$, with $|\delta\xi ^{m}(t)| \ll1$. Writing the corresponding perturbation of $\widehat {\psi}(\xi,t)$ as $\widehat{\psi}(\xi,t) = \widehat{\psi}(\xi) + \delta\widehat{\psi}(\xi,t)$ we find

36 $$ \delta\widehat{\psi}(\xi,t) = c^{2} \sum_{m \in \mathbb{Z}} \int _{0}^{\infty} \mathrm {d}s \eta(s) w' \bigl(\big|c(s-\xi) + c m \varDelta\big|\bigr) \delta\xi^{m} (t-s) . $$

It remains to determine the relationship between $\delta\xi^{m}(t)$ and the perturbations of the shape of the travelling wave. In Appendix B we show that we can relate $\delta\xi^{m}(t)$ to the deviation in the voltage, denoted by $\delta\widehat{V}(m \varDelta,t)$, via the simple relationship

37 $$ \delta\xi^{m}(t) = - \frac{\delta\widehat{V}(m \varDelta,t)}{V_{\xi}(m \varDelta_{-})} . $$

Thus for solutions of the form $\delta\widehat{V}(\xi,t) = \delta \widehat{V}(\xi) \mathrm {e}^{\lambda t}$, $\delta\widehat{V}(\xi) = \delta\widehat{V}(\xi+\varDelta)$ we find $\delta\widehat{\psi}(\xi,t) = \delta\widehat{\psi}(\xi;\lambda ) \mathrm {e}^{\lambda t}$, with $\delta\widehat{\psi}(\xi;\lambda) =\delta\widehat{V}(0) f(\xi ;\lambda)$, and

38 $$ f(\xi;\lambda) = -\frac{c^{2}}{V_{\xi}(0)} \sum_{m \in \mathbb{Z}} \int _{0}^{\infty} \mathrm {d}s \eta(s) w' \bigl(\big|c(s-\xi)+cm\varDelta\big|\bigr) \mathrm {e}^{-\lambda s}. $$

Returning to the more abstract setting given by Eq. (11) we linearise around the travelling wave by setting $\widehat{X}(\xi,t) = Q(\xi) + \delta\widehat{X}(\xi) \mathrm {e}^{\lambda t}$, with $\delta \widehat{X}(\xi)=\delta\widehat{X}(\xi+\varDelta)$. This yields the variational equation

39 $$ \frac{\mathrm {d}}{\mathrm {d}\xi} \delta\widehat{X}(\xi) = A(\xi;\lambda )\delta\widehat{X}( \xi) + \delta\widehat{\Psi}(\xi;\lambda), \quad\delta\widehat{\Psi}(\xi;\lambda) = \frac{g_{\text{syn}} g_{\text{l}}^{-1}}{\tau} \delta\widehat{\psi}(\xi;\lambda) \begin{bmatrix} 1 \\0 \end{bmatrix} , $$

where $A(\xi;\lambda) = A(Q(\xi)) -\lambda I_{2}$ (and we use the argument of *A* to emphasise that it depends on position along the periodic orbit). We may write the solution to (39) in much the same way as for the periodic orbit problem given by (18), namely with the use of a variation of parameters formula, matrix exponentials and (38):

40 $$ \delta\widehat{X}(\xi) = \textstyle\begin{cases} G_{\text{R}}(\xi;\lambda) \delta\widehat{X}(0) & 0 \leq\xi< \tau _{\text{R,}} \\ G_{0}(\xi-\tau_{\text{R}};\lambda) \delta\widehat{X}(\tau_{\text{R}}) \\\quad{}+ \int_{\tau_{\text{R}}}^{\xi} \mathrm {d}\xi' G_{0}(\xi-\xi';\lambda) J f(\xi';\lambda) \delta\widehat{X}(0) & \tau_{\text{R}} \leq\xi< \tau_{\text{R}} +\xi_{1}, \\ G_{+}(\xi-(\tau_{\text{R}} +\xi_{1});\lambda) \delta\widehat{X}(\tau _{\text{R}} +\xi_{1}) \\ \quad{}+\int_{\tau_{\text{R}} +\xi_{1}}^{\xi} \mathrm {d}\xi' G_{+}(\xi-\xi';\lambda) J f(\xi';\lambda) \delta\widehat{X}(0) & \tau_{\text{R}} +\xi_{1} \leq\xi< \varDelta. \end{cases} $$

Here $G_{\mu}(\xi;\lambda)=\exp([A_{\mu}- \lambda I_{2} ] \xi)$ and

41 $$ J= \frac{g_{\text{syn}} g_{\text{l}}^{-1}}{\tau} \begin{bmatrix} 1 & 0 \\ 0 & 0 \end{bmatrix} . $$

However, the evolution of perturbations through the switching manifolds $V=V_{\pm}$, the firing threshold $V=V_{\text{th}}$ and the release from the refractory state requires care, since in all these cases there is a jump in the Jacobian. The theory of nonsmooth dynamical systems gives a prescription for handling this using so-called *saltation matrices* dating back to the work of Aizerman and Gantmakher in the 1950s [41]. For a more recent perspective we recommend the paper by Leine et al. [42] and the book by di Bernardo et al. [43], particularly in engineering applications, and the paper by Coombes et al. [44] for applications in neuroscience. The $2 \times2$ saltation matrices for handling switching, firing, and refractoriness are constructed in Appendix C and denoted $K_{\text{switch}}$, $K_{\text{fire}}$, and $K_{\text{ref}}$, respectively. In essence they map perturbations through the region of nonsmooth behaviour to give $\delta\widehat{X}(0_{+}) = K_{\text{fire}} \delta \widehat{X}(0)$, $\delta\widehat{X}({\tau_{\text{R}}}_{+}) = \delta \widehat{X}({\tau_{\text{R}}})+ K_{\text{ref}} \delta\widehat {X}(0)$, and $\delta\widehat{X}((\tau_{\text{R}}+\xi_{1})_{+}) = K_{\text{switch}} \delta\widehat{X}(\tau_{\text{R}}+\xi_{1})$. The saltation matrices are given explicitly by $K_{\text{switch}} = I_{2}$ and

42 $$ \begin{aligned} K_{\text{fire}} &= \begin{bmatrix} 0 & 0 \\ (n_{\text{h},\xi}(0_{+})-n_{\text{h},\xi }(0_{-}))/{V_{\xi}(0_{-})} & 1 \end{bmatrix} , \\ K_{\text{ref}}& = \begin{bmatrix} {V_{\xi}({\tau_{\text{R}}}_{+})}/{V_{\xi}(0_{-})} & 0 \\ 0 & 0 \end{bmatrix} .\end{aligned} $$

If we now introduce the function $\mathcal{F}_{\mu}(\xi,\xi_{0};\lambda)$:

43 $$ \mathcal{F}_{\mu}(\xi,\xi_{0};\lambda) = \int_{\xi_{0}}^{\xi} \mathrm {d}\xi' G_{\mu}\bigl(\xi-\xi';\lambda\bigr) J f\bigl(\xi';\lambda\bigr) , \quad\mu\in\{0, +,-\}, $$

then Eq. (40) may be used to generate the perturbation after one period as $\delta\widehat{X}(\varDelta) = \varGamma(\lambda,\varDelta) \delta\widehat{X}(0)$, where

44 $$\begin{aligned} \varGamma(\lambda, \varDelta) ={}& \mathcal{F}_{+} (\varDelta, \tau_{\text{R}}+ \xi_{1};\lambda) \\ &+ G_{+}\bigl(\varDelta-(\tau_{\text{R}}+\xi_{1});\lambda\bigr) K_{\text{switch}} \bigl[ \mathcal{F}_{0} (\tau_{\text{R}}+ \xi_{1},\tau_{\text{R}};\lambda) \\ & + G_{0}(\xi_{1};\lambda) \bigl[G_{\text{R}} (\tau_{\text{R}};\lambda) K_{\text{fire}} + K_{\text{ref}}\bigr] \bigr] .\end{aligned} $$

Enforcing that perturbations be *Δ*-periodic (i.e. $\delta\widehat{X}(\varDelta) = \delta\widehat{X}(0)$) we obtain the spectral condition $\mathcal{E}(\lambda, \varDelta) = 0$ where

45 $$ \mathcal{E}(\lambda, \varDelta) = \big|\varGamma(\lambda, \varDelta) -I_{2}\big| . $$

We identify (45) as the Evans function for the periodic wave. To determine (43) in a computationally useful form we use a Fourier representation to represent (38) (cf. (20) from (19)) as $f(\xi ;\lambda) = \sum_{p \in \mathbb{Z}} f_{p}(\lambda) \exp(- 2 \pi i p \xi /\varDelta)$ where

46 $$ f_{p}(\lambda) = -\frac{1}{V_{\xi}(0)} \frac{2 \pi}{\varDelta^{2}} ip \widetilde{\eta}(-i \lambda- 2 \pi p/\varDelta) \widetilde{w}\bigl(2 \pi p /(c \varDelta)\bigr) , $$

for $\operatorname{Re} (\lambda+\alpha) >0$. Then in a similar way to the construction of (26) we find the useful representation for (43) as

47 $$ \mathcal{F}_{\mu}(\xi,\xi_{0};\lambda) = \sum _{p} f_{p}(\lambda) P_{\mu}\operatorname{diag} \bigl( S_{\mu}^{+}(\xi,\xi_{0};\lambda), S_{\mu}^{-}(\xi ,\xi_{0};\lambda)\bigr) P_{\mu}^{-1} J , $$

where

48 $$ S_{\mu}^{\pm}(\xi,\xi_{0};\lambda) = \frac{\mathrm {e}^{(\lambda_{\mu}^{\pm}-\lambda)(\xi-\xi_{0})} \mathrm {e}^{-2 \pi i p \xi_{0}/\varDelta} - \mathrm {e}^{-2 \pi i p \xi/\varDelta}}{\lambda_{\mu}^{\pm}-\lambda+2 \pi i p/\varDelta} . $$

**Fig. 10:**
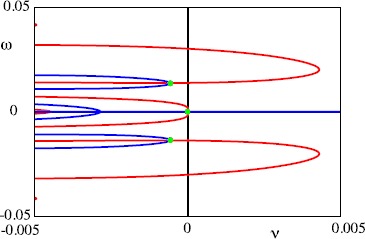
Zeros of the Evans function (45) with $\varDelta= 460$. These occur at the intersection (*green dots*) of $\mathcal{G}(\nu, \omega)=0$ (*red curve*) and $\mathcal{H}(\nu, \omega)=0$ (*blue curve*) where $\mathcal{G}$ is the real part of ${\mathcal{E}}$ whereas $\mathcal {H}$ is the imaginary part. Here we can see that all the eigenvalues, except the zero eigenvalue, have negative real part, so that the periodic wave is stable. Parameters as in Fig. 5

**Fig. 11:**
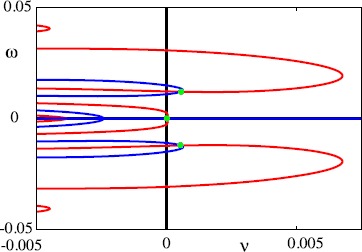
Zeros of the Evans function (45) with $\varDelta= 470$. These occur at the intersection (*green dots*) of $\mathcal{G}(\nu, \omega)=0$ (*red curve*) and $\mathcal{H}(\nu, \omega)=0$ (*blue curve*) where $\mathcal{G}$ is the real part of ${\mathcal{E}}$ whereas $\mathcal{H}$ is the imaginary part. Here we see a complex conjugate pair of eigenvalues with positive real part, so that the periodic wave is unstable. Parameters as in Fig. 5

The eigenvalues of the spectral problem can be practically constructed by considering the decomposition $\lambda= \nu+ i \omega$ and simultaneously solving the pair of equations $\mathcal{G}(\nu, \omega )=0$ and $\mathcal{H}(\nu, \omega)=0$, where $\mathcal{G}(\nu, \omega)=\operatorname{Re} \mathcal{E}(\nu+ i \omega, \varDelta)$ and $\mathcal{H}(\nu, \omega)=\operatorname{Im} \mathcal{E}(\nu+ i \omega, \varDelta)$, subject to the constraint $\nu+\alpha>0$. Figures 10 and 11 show the zero level sets of $\mathcal {G}$ and $\mathcal{H}$ in the $(\nu,\omega)$ plane for two different points on the dispersion curve of Fig. 9. The intercepts when $\nu+\alpha>0$ provide the zeros of the Evans function and here highlight clearly that, for *Δ* sufficiently large, the zeros of the Evans function can cross to the right-hand complex plane signalling a wave instability.

Figure 10 also shows that there is always a zero eigenvalue. It is simple to establish the persistence of this zero under parameter variation. Differentiating (18) with respect to *ξ* gives

49 $$ \frac{\mathrm {d}}{\mathrm {d}\xi} \frac{\mathrm {d}Q}{\mathrm {d}\xi} = A \frac{\mathrm {d}Q}{\mathrm {d}\xi} + \frac{\mathrm {d}}{\mathrm {d}\xi} \widehat{\Psi}(\xi). $$

From (38) and (19) we may establish that

50 $$ \delta\widehat{\psi}(\xi;0) = \frac{\delta\widehat{V}(0)}{V_{\xi}(0)} \frac{\mathrm {d}}{\mathrm {d}\xi} \widehat{ \psi}(\xi) . $$

Hence for $\lambda=0$ we see that a solution to (39) is the eigenfunction

51 $$ \delta\widehat{X}(\xi) = \frac{\mathrm {d}Q}{\mathrm {d}\xi} , $$

as expected from translation invariance of the system (so that a perturbation tangential to the travelling wave orbit is neutrally stable).

## Discussion

This paper is motivated by recent work in computational neuroscience that has highlighted rebound firing as a mechanism for wave generation in spiking neural networks that can underlie the formation of grid cell firing fields in MEC [1]. We have presented a simple spiking model with inhibitory synaptic connections and an $I_{\text{h}}$ current that can generate smoothly propagating activity waves via post-inhibitory rebound. These are qualitatively of the type observed in previous computational studies [36, 37], yet are amenable to an explicit mathematical analysis. This is possible because we have chosen to work with piecewise linear discontinuous models, and exploited methodologies from the theory of nonsmooth systems. In particular we have exploited the linearity of our model between events (for firing, switching, and release from a refractory state) to construct periodic solutions in a travelling wave frame. To assess the stability of these orbits we have treated the propagation of perturbations through event manifolds using saltation operators. Using this we have constructed dispersion curves showing a wide range of stable periods, with a maximum period controlled by the time-scale of the rebound current. This gives further credence to the idea that the change in grid field scale along the dorsal-ventral axis of the MEC is under local control by HCN channels.

A number of natural extensions of the work in this paper suggest themselves. We briefly outline them here, and in no particular order. For simplicity we have focussed on the analysis of waves in a homogeneous model with only one spatial dimension. The analysis of the corresponding travelling waves, with hexagonal structure, in two spatial dimensions is more challenging, though is an important requirement for a complete model of grid cell firing. Moreover, the assumption of homogeneity should be relaxed. In this regard it would be of interest to understand the effects of a heterogeneity in the time constants (for voltage response, synaptic time-scale, and the time-scale of the $I_{\text{h}}$ current) on the properties of spatio-temporal patterns. Furthermore, it would be biologically more realistic to consider two sets of inhibitory interneurons, as in [1, 28, 30]. As well as depending on the $I_{\text{h}}$ current, grid field spacing changes as a function of behavioural context. This is believed to occur through the activation of neuromodulators [32], and simple regulation of our $I_{\text{h}}$ current model would allow a systematic study of this, even before considering the more important issue of structured input. The work in this paper has focussed on spontaneous pattern formation that occurs in the absence of such input. Given the importance of the head-direction input for driving grid cell firing fields it would be natural to consider a mathematical analysis for the case with $I_{\text{hd}} \neq0$. For the standard Fuhs–Touretzky mechanism of inducing firing patterns this would further require the treatment of an anisotropic interaction kernel with a dependence on a head-direction preference map. One way to address this would be via a perturbation theory around the limiting case treated in this paper, and use this to calculate a tissue response parametrised by the animal’s speed and direction of motion. The same methodology would also allow an investigation of phase precession during a grid field traversal. Our simulations have also shown the possibility of ‘lurching waves’ and it would be interesting, at least from a mathematical perspective, to analyse their properties (speed and stability) and compare them to the co-existing smoothly propagating waves. It would further be pertinent in this case to pay closer attention to any possible nonsmooth bifurcations that could give rise to wave instabilities, such as grazing bifurcations. Finally we note that grid cells are grouped in discrete modules with common grid spacing and orientation [20]. It has recently been suggested that coupling between modules or via feedback loops to the hippocampus may help to suppress noise and underpin a robust code (with a large capacity) for the representation of position [45]. Another extension of the work in this paper would thus be to consider the dynamics of networks of interacting modules, paying closer attention to the details of MEC microcircuitry [46].

### Electronic Supplementary Material

Below are the links to the electronic supplementary material.

Code for 2D simulations. Code written in C^++^/CUDA programming language. Equipment requirements: Nvidia CUDA drivers and Nvidia graphics card with compute capability ≥3.5. The Armadillo C^++^ package must be installed. Run commands: $ cmake . $ make $./SpatialNavigationCUDA. (zip)

Movie for 2D patterned state evolution. A movie of the voltage component in a planar spiking neural field model with an $I_{\mathrm{h}}$ current. Here we see the emergence of a rich patterned state. Parameters as in Fig. 2 and the system is initialised with a localised region of hyperpolarisation. Activity spreads from this initial state generating spatio-temporal patterns that interact in a complicated fashion to generate hexagonal structures. (m4v)

## Figures and Tables

**Algorithm 1:**
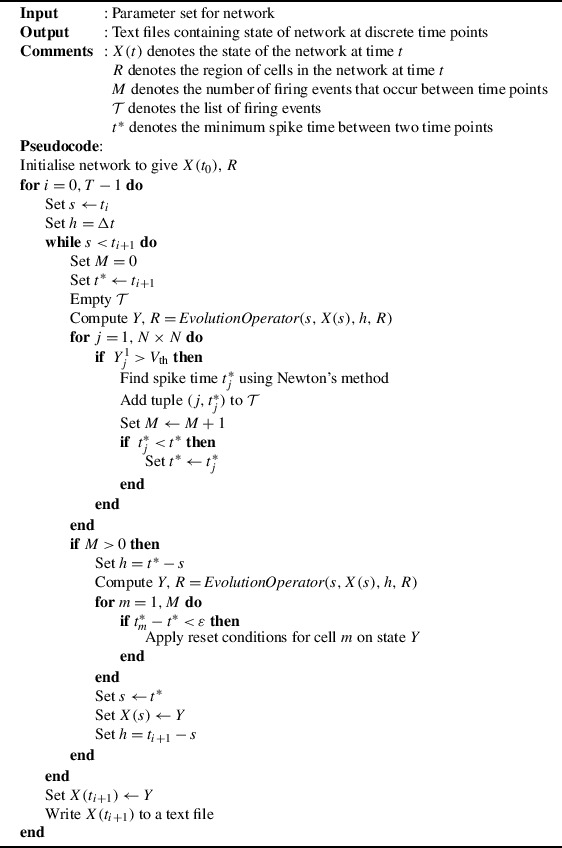
Evolution of 2D network

**Algorithm 2:**
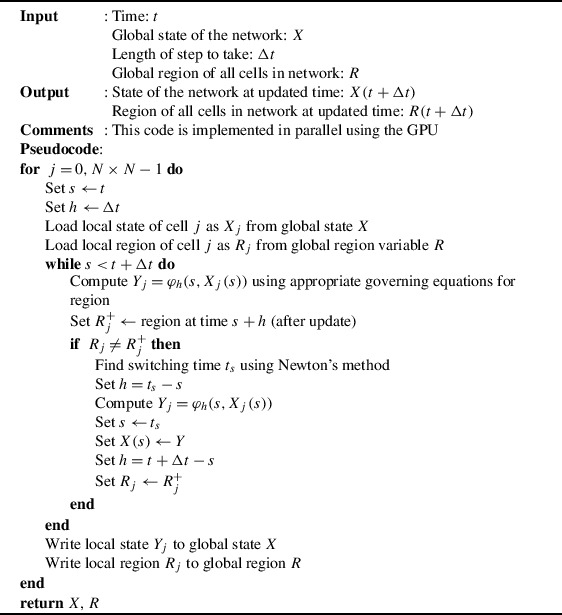
EvolutionOperator

## Appendix A: Numerical Scheme

Here, we describe the numerical scheme that we have developed to evolve the spiking neural field model. Given the large computational overheads for simulating the latter we have focussed on implementing the algorithm on a GPU system. In essence, we exploit the piecewise linear nature of the dynamics to obtain trajectories in closed form and then use a root-finding scheme to find the timings of events (switching, firing or refractory). The dynamics is updated at an event (generating synaptic currents and resetting at firing events, changing the gating dynamics at switching events, and releasing from reset at refractory events), and the process repeated. For clarity we describe our approach for two spatial dimensions as it is easily adapted to treat just one spatial dimension.

### A.1 Algorithm

We solve the system over a 2D discretised grid of size $L \times L$ with *N* (numerical) cells in each spatial dimension and enforce periodic boundary conditions. Cells are equi-spaced with a spatial separation of $\varDelta x = 2L/N$ and the state of the cells in the network are evaluated over $T+1$ time steps, discretising time as $t_{i} = i \Delta t$, $i=0,\dots,T$. To evaluate these states, we analytically integrate (11) to provide a closed-form expression for the trajectory of the system. Our closed-form expression allows us, for cell *j*, to write its state at time $t_{i+1}$ as a function of its state at time $t_{i}$:

$$X_{j}(t_{i+1}) = \varphi_{\Delta t} \bigl(t_{i},X_{j}(t_{i}) \bigr), $$

where $\varphi_{h}$ is the evolution operator acting over a time *h*. Note that this expression assumes that no firing events have occurred between $t_{i}$ and $t_{i+1}$, but can account for switching events, as detailed below. We shall describe later how the algorithm handles firing events.

For convenience, we shall divide the state space of an individual cell into three regions, based on the instantaneous value of *V*: Region I, with $V\leq V_{-}$; Region II, with $V_{-}< V\leq V_{+}$; and Region III, with $V>V_{+}$. In addition, we define Region IV to be that where the cell is in the refractory state (recall that a cell is in the refractory state at time *t* if $T^{m-1}\geq t+\tau_{\mathrm{R}}$, where $T^{m-1}< t$ is the last spiking time of that cell). In each of these regions, the equation governing the dynamics for the gating variable $n_{\mathrm{h}}$ is different, and this needs to be reflected in our algorithm.

At a given time *t*, each cell in the network is in precisely one of the regions outlined in the preceding paragraph. We store a global array that tracks which region each of the cells is in and use this to select the appropriate equation to integrate, based on those presented in (13). Over an interval Δ*t*, the region of a subset of the cells may change as the local voltage variable, *V*, crosses switching manifolds. Note that the continuity of trajectories restricts which transitions between regions are possible (for example, a transition between Region I and Region III is not permitted). Our evolution operator $\varphi_{h}$ must account for this.

We identify where switching events occur by comparing the region of all of the cells before and after the operator $\varphi_{\Delta t}$ is applied. Where switching events have occurred, we locate the switching time for cell *j* by searching for roots of the transcendental equation $X_{j}^{1}(s) - V_{x} = 0$, $t_{i} \leq s < t_{i+1}$ using Newton’s method, where the superscript denotes that we only consider the *V* component of the state vector $X_{j}$ and $V_{x}$ is the switching manifold that has been crossed. We remark that the Fréchet derivatives for use in Newton’s method are obtainable in closed form, and so the application of Newton’s method does not rely on numerical finite difference approximations for these derivatives.

After the switching time, *s*, has been found, the state of the cell is updated via $X_{j}(s) = \varphi_{\Delta s}(t_{i},X_{j}(t_{i}))$, where $\Delta s = s-t_{i}$, according to the governing equation for that region. Following this, the regional variable is updated to reflect the fact that the cell has transitioned to a new region. Finally, the remainder of the update step is taken, according to $X_{j}(t_{i+1}) = \varphi_{\Delta s^{*}} (s,X_{j}(s))$, where $\Delta s^{*} = t_{i+1}-s$, by integrating the governing equation for new region. It is possible that between times *s* and $t_{i+1}$, the cell passes through other switching manifolds, in which case, the procedure for identifying switching times and updating the region variable is repeated as many times as necessary.

Cells in Region IV are in the refractory state, governed by (12) and (14), in which the voltage variable is held fixed at $V=V_{\mathrm{r}}$ for a total duration of length $\tau_{\mathrm{R}}$. To account for this, we introduce for each cell, a counter that tracks how much time a cell has spent in the refractory period. Following a firing event, the spiking cell enters the refractory state, whereupon this counter is reset to zero (and the cell’s region is changed to Region IV). The cell will remain in the refractory state until the counter reaches $\tau_{\mathrm{R}}$. At this time, the region variable for that cell is updated to the appropriate value, based on the location of $V_{\mathrm{r}}$ relative to $V_{-}$ and $V_{+}$. For computational efficiency, we can take advantage of the fact that *V* is unchanging in the refractory period by replacing the voltage dynamics with $\dot{V}=1$, thus letting *V* implement our refractory time counter in Region IV. Thus, if the cell spikes at time $T^{m}$, we have $V(T^{m})=0$ upon entering Region IV. To ensure continuity of solutions upon exiting the refractory period, we must also ensure to set $V(T^{m}+\tau_{\mathrm{R}}) = V_{\mathrm{r}}$.

Since cells are independent of one another outside of firing times, this above computation to update the state of the system can be performed in parallel across the GPU architecture in the absence of firing events. Where firing events occur, we must ensure that we correctly update the state of the network at the firing time to reflect the coupling in the network. As for the switching times, we determine firing times by searching for roots of the transcendental equation $X_{j}^{1}(s) - V_{\text{th}} = 0$, $t_{i} \leq s < t_{i+1}$ using Newton’s method. We then amalgamate all firing events between times $t_{i}$ and $t_{i+1}$ and find the minimum firing time, $t^{*}$, of this list. The states of all cells are updated to this time from $t_{i}$:

$$X^{-}(t_{*}) = \varphi_{\Delta t^{*}} \bigl( t_{i}, X(t_{i}) \bigr), $$

where $\Delta t^{*} = t^{*}-t_{i}$ and the superscript denotes that this corresponds to the state of the network in the limit as *t* approaches $t^{*}$ from the left. Following this, the reset conditions are applied across the entire domain. Our approach allows for simultaneous firing events to occur by also applying reset conditions for all events that occurred with the time interval $[t^{*},t^{*}+\varepsilon)$, where *ε* is a small positive real number. After the reset conditions have been applied, the state of the network is updated again as

$$X(t_{i+1}) = \varphi_{\Delta t^{*}_{c}} \bigl( t_{*}, X^{+}(t_{*}) \bigr), $$

where $\Delta t^{*}_{c} = t_{i+1} - t^{*}$ and the superscript denotes that the state is evaluated after the reset conditions have been applied. Note that during the completion of the timestep, we must again check to see if other cells have reached threshold, and apply reset conditions as necessary. At the completion of a timestep, the state of the network is saved and the state at the next time step is computed.

We note that a further improvement in computational efficiency can be achieved in the limit of high gain for the kernel function (7). Namely in the limit $\beta\rightarrow\infty$ the bump function becomes a Top-hat function:

52 $$ w(r) = \textstyle\begin{cases} w_{0} , & r\leq\sigma, \\ 0 , & r>\sigma. \end{cases} $$

Thus, cells are only coupled with a single strength $w_{0}$, over a finite distance, *σ*, and we need only consider the behaviour of other cells within this distance when applying reset conditions.

### A.2 Implementation

To take full advantage of the parallel capabilities of GPUs, we must sensibly organise the processes associated with simulating the network through the construction of appropriate kernels to best to perform tasks in parallel. In addition to this, the memory associated with state variables must be managed appropriately given that memory access can present a significant bottleneck during GPU-based computations.

As discussed in the preceding section, the evolution operator $\varphi _{h}$ has to account for the differing functional forms of the governing equations as *V* crosses thresholds. This is done by using Newton’s method to find the times of any crossings and then by ‘patching’ together solution orbits on either side of the threshold crossing time. Associated with Newton’s method is a tolerance for finding the crossing times, which must be specified along with other parameters. In our implementation, we take advantage of the double precision capability of our GPU. Since we are using closed-form solutions for the orbits of our system, the only source of numerical error other than machine error arises due to the tolerance selected when computing the threshold crossing times for *V*. In all of our simulations, we prescribe this tolerance to be 10^−10^ so that we have precise specification of these times.

During an update step, there may be multiple cells that reach the firing threshold at differing times. To preserve the correct dynamics in spite of this, we need to update the state of the network to the minimum of this set of firing times. To identify this, we define a variable that stores the minimum firing time across the network, which is updated by the subset of spiking cells as firing events are found. Without intervention, this procedure can lead to *race* conditions, in which one spiking cell will query the stored value of the minimum firing time (to compare with its own), whilst another spiking cell is replacing that same value. Race conditions mean that we cannot guarantee that the value stored in our firing time variable truly represents the minimum over all firing events. To address this problem, we use *atomic* events, which allow only one cell to read from and write to the stored firing time variable at once. In this way, we are guaranteed to find the true minimum across the set of firing times.

In Algorithms 1 and 2, we detail how to evolve the dynamics of the 2D network. Note that all of the *for* loops, and the use of $\varphi _{h}$ to update the state of the network are performed in parallel using the GPU. The full code for our simulations, written in the C^++^/CUDA programming language, is available in the supplementary material.

## Appendix B: Perturbations at Switching Conditions

The model undergoes *switching* in its dynamics as the voltage variable passes through the values *V*+, $V_{-}$, and $V_{\text{th}}$. With the introduction of a set of indicator functions $h(X(\xi,t);\mu ) = V(\xi,t)-V_{\mu}$, where $\mu\in\{+,-,\text{th} \}$ we can define the travelling wave coordinate values at which these switching events occur according to $h(X(\xi,t);\mu) = 0$. Now suppose that we have two trajectories: an unperturbed trajectory $X(\xi,t)=(V(\xi ,t),n_{\text{h}}(\xi,t))$ and a perturbed trajectory $\widetilde {X}(\xi,t)$ such that $\delta{X}(\xi,t) = \widetilde{X}(\xi ,t)-X(\xi,t)$, with *δX* small. Moreover, let us consider the unperturbed trajectory to pass through the switching manifold when $\xi = \xi^{m}(t)$, $m \in \mathbb{Z}$. Similarly we shall consider the perturbed trajectory to switch when $\xi= \widetilde{\xi}^{m}(t) = {\xi}^{m}(t) +\delta\xi^{m}(t)$. The indicator function for the perturbed trajectory may be Taylor expanded as (suppressing the dependence on *μ* for clarity):

53 $$\begin{aligned}[b] h\bigl(\widetilde{X}\bigl(\widetilde{\xi}^{m},t\bigr)\bigr) ={}& h\bigl( \widetilde{X}\bigl({\xi}^{m} +\delta\xi^{m}\bigr),t\bigr) \\ ={}& h \bigl(X\bigl({\xi}^{m} +\delta\xi^{m},t\bigr)+\delta X\bigl({ \xi}^{m} +\delta\xi^{m},t\bigr)\bigr) \\ \simeq{}& h\bigl(X\bigl({\xi}^{m},t\bigr) +X_{\xi}\bigl({ \xi}^{m}_{-},t\bigr)\delta\xi^{m}\bigr)\\ &+\nabla_{X} h \bigl(X\bigl({\xi}^{m} +\delta\xi^{m},t\bigr)\bigr) \cdot \delta X\bigl({\xi}^{m} +\delta\xi ^{m},t\bigr) \\ \simeq{}& h\bigl(X\bigl({\xi}^{m},t\bigr)\bigr) + \nabla_{X} h\bigl(X\bigl({\xi}^{m},t\bigr)\bigr) \cdot X_{\xi}\bigl({\xi }^{m}_{-},t\bigr)\delta\xi^{m}\\ & + \nabla_{X} h\bigl(X \bigl({\xi}^{m},t\bigr)\bigr) \cdot\delta X\bigl({\xi }^{m},t \bigr) .\end{aligned} $$

Here we have introduced the notation $X({\xi}^{m}_{\pm},t) = \lim_{\epsilon\searrow0} X({\xi}^{m} \pm\epsilon,t)$, to make sure that the partial derivative in *ξ* is well defined. Using the fact that $h(\widetilde{X}(\widetilde{\xi }^{m},t))=0=h({X}({\xi}^{m},t))$ we obtain

54 $$ \nabla_{X} h\bigl({X}\bigl({\xi}^{m},t\bigr)\bigr) \cdot \bigl[ \delta X\bigl({\xi}^{m},t\bigr) + X_{\xi}\bigl({ \xi}^{m}_{-},t\bigr)\delta\xi^{m} \bigr]=0 . $$

Using the result that $\nabla_{X} h(X;\mu) = (\partial_{V},\partial _{n_{\text{h}}}) (V-V_{\mu})=(1,0)$ the above can be re-arranged to give the perturbation in the switching coordinate in terms of the difference between the perturbed and unperturbed trajectories as

55 $$ \delta\xi^{m}(t) = - \frac{\delta V(\xi^{m},t)}{V_{\xi}(\xi^{m}_{-},t)} . $$

## Appendix C: Saltation Matrices

Saltation matrices allow us to handle any *jumps* in the system (or its linearisation) when it changes from one dynamical regime to another. As well as occurring at the switching manifolds this also happens when the voltage is *unclamped* and released from the refractory state. Using the notation of Appendix B let us first consider the deviation between the two trajectories at a switching event defined by $\xi=\xi _{\text{s}}$, with a set of perturbed switching events at $\xi=\xi _{\text{s}} + \delta\xi$, as

56 $$\begin{aligned}[b] \delta X(\xi_{\text{s}}+\delta\xi,t) &= \widetilde{X}(\xi_{\text{s}}+\delta\xi,t) - {X}(\xi_{\text{s}}+\delta\xi,t) \\ &\simeq\widetilde{X}(\xi_{\text{s}},t) + \widetilde{X}_{\xi}(\xi _{\text{s}},t) \delta\xi-\bigl[{X}(\xi_{\text{s}},t) + {X}_{\xi}(\xi_{\text{s}},t) \delta\xi\bigr] \\ &=\delta{X}(\xi_{\text{s}},t) +\bigl[\widetilde{X}_{\xi}( \xi_{\text{s}},t)-{X}_{\xi}(\xi_{\text{s}},t)\bigr]\delta \xi.\end{aligned} $$

If $\delta\xi>0$ then the unperturbed trajectory will already have transitioned through the switch (from below), in which case the two trajectories are governed by different dynamics. A similar argument holds for $\delta\xi<0$. Thus we may write

57 $$ \delta X(\xi_{\text{s}}+\delta\xi,t) \simeq\delta X(\xi_{\text{s}},t) + \bigl[{X}_{\xi}({\xi_{\text{s}}}_{-},t)-{X}_{\xi}({ \xi_{\text{s}}}_{+},t) \bigr] \delta\xi. $$

Combining (55) and (57) gives

58 $$ \begin{bmatrix} \delta V(\xi_{\text{s}}+\delta\xi,t) \\ \delta n_{\text{h}}(\xi_{\text{s}}+\delta\xi,t) \end{bmatrix} = \begin{bmatrix} \delta V(\xi_{\text{s}},t) \\ \delta n_{\text{h}}(\xi_{\text{s}},t) \end{bmatrix} - \frac{\delta V(\xi_{\text{s}},t)}{V_{\xi}({\xi_{\text{s}}}_{-},t)} \begin{bmatrix} V_{\xi}({\xi_{\text{s}}}_{-},t)-V_{\xi}({\xi_{\text{s}}}_{+},t)\\ n_{\text{h},\xi}({\xi_{\text{s}}}_{-},t)-n_{\text{h},\xi}({\xi_{\text{s}}}_{+},t) \end{bmatrix} . $$

We may write the above in matrix form as

59 $$ \begin{bmatrix} \delta V(\xi_{\text{s}}+\delta\xi,t) \\ \delta n_{\text{h}}(\xi_{\text{s}}+\delta\xi,t) \end{bmatrix} = \begin{bmatrix} 1 - \frac{V_{\xi}({\xi_{\text{s}}}_{-},t)-V_{\xi}({\xi_{\text{s}}}_{+},t)}{V_{\xi}({\xi_{\text{s}}}_{-},t)} & 0 \\ -\frac{n_{\text{h},\xi}({\xi_{\text{s}}}_{-},t)-n_{\text{h},\xi }({\xi_{\text{s}}}_{+},t)}{V_{\xi}({\xi_{\text{s}}}_{-},t)} & 1 \end{bmatrix} \begin{bmatrix} \delta V(\xi_{\text{s}},t) \\ \delta n_{\text{h}}(\xi_{\text{s}},t) \end{bmatrix} , $$

or equivalently as $\delta X(\xi_{\text{s}}+\delta\xi,t)=K(\xi_{\text{s}}) \delta X(\xi_{\text{s}},t)$ with

60 $$ K(\xi_{\text{s}}) = \begin{bmatrix} {V_{\xi}({\xi_{\text{s}}}_{+},t)}/{V_{\xi}({\xi_{\text{s}}}_{-},t)} & 0 \\ (n_{\text{h},\xi}({\xi_{\text{s}}}_{+},t)-n_{\text{h},\xi}({\xi _{\text{s}}}_{-},t))/{V_{\xi}({\xi_{\text{s}}}_{-},t)} & 1 \end{bmatrix} . $$

Now since the voltage is clamped immediately after a firing event (so that ${V_{\xi}({\xi_{\text{s}}}_{+},t)}=0$) and the dynamics for $n_{\text{h}}$ jumps (since it depends on *V* which is discontinuously reset from $V_{\text{th}}$ to $V_{\text{r}}$) then the saltation matrix for firing is given by

61 $$ K_{\text{fire}}(\xi_{\text{s}}) = \begin{bmatrix} 0 & 0 \\ (n_{\text{h},\xi}({\xi_{\text{s}}}_{+},t)-n_{\text{h},\xi}({\xi_{\text{s}}}_{-},t))/{V_{\xi}({\xi_{\text{s}}}_{-},t)} & 1 \end{bmatrix} . $$

At a switching event whenever $V=V_{\pm}$ we note that the voltage and gating dynamics are both continuous. Thus the saltation matrix for switching is given simply by $K_{\text{switch}}(\xi_{\text{s}})=I_{2}$, namely there is no effect.

The use of saltation matrices to propagate perturbations through the refractory state is a little more subtle than through switching and firing events, since the former occur over a finite time-scale $\tau_{\text{R}}$ whilst the latter are instantaneous. In this case the perturbation *δξ* at a firing event $\xi=\xi_{\text{f}}$ is propagated for a time $\tau_{\text{R}}$ before a new dynamical regime is encountered. From (55) $\delta\xi= - \delta V(\xi_{\text{f}},t)/V_{\xi}({\xi_{\text{f}}}_{-},t)$. Setting $\xi_{\text{s}}=\xi_{\text{f}}+\tau_{\text{R}}$ and combining the above with (57) gives

62 $$ \delta X(\xi_{\text{s}}+\delta\xi,t) \simeq\delta X(\xi_{\text{s}},t) - \frac{\delta V(\xi_{\text{f}},t)}{V_{\xi}({\xi_{\text{f}}}_{-},t)} \begin{bmatrix} V_{\xi}({\tau_{\text{R}}}_{-},t)-V_{\xi}({\tau_{\text{R}}}_{+},t)\\ n_{\text{h},\xi}({\tau_{\text{R}}}_{-},t)-n_{\text{h},\xi}({\tau _{\text{R}}}_{+},t) \end{bmatrix} . $$

Hence we see that $\delta X({\xi_{\text{s}}}_{+},t) = \delta X(\xi_{\text{s}},t) + K_{\text{ref}}(\xi_{\text{s}}) \delta X(\xi_{\text{f}},t)$ where

63 $$ K_{\text{ref}}(\xi_{\text{s}}) = \begin{bmatrix} {V_{\xi}({\xi_{\text{s}}}_{+})}/{V_{\xi}({\xi_{\text{f}}}_{-})} & 0 \\ 0 & 0 \end{bmatrix} . $$

Here we have used the fact that ${V_{\xi}({\xi_{\text{s}}}_{-},t)}=0$ (since the system is in its refractory state), and that the dynamics for $n_{\text{h}}$ is continuous at $\xi=\xi_{\text{s}}$.
